# The BDNF Val66Met polymorphism serves as a potential marker of body weight in patients with psychiatric disorders

**DOI:** 10.3934/Neuroscience.2024012

**Published:** 2024-06-24

**Authors:** Yinghua Zhang, Xinyue Wei, Wenhao Zhang, Feng Jin, Wenbo Cao, Mingjin Yue, Saijun Mo

**Affiliations:** 1 Department of Pathophysiology, School of Basic Medical Sciences, Zhengzhou University, Zhengzhou, China; 2 Collaborative Innovation Center of Henan Province for Cancer Chemoprevention, Zhengzhou, China; 3 State Key Laboratory of Esophageal Cancer Prevention and Treatment, Zhengzhou, China; 4 Henan Tianxing Education and Media Company, Limited, Zhengzhou, China

**Keywords:** BDNF, Val66Met polymorphism, body weight, psychiatric disorders, marker

## Abstract

Brain-derived neurotrophic factor (BDNF) is a predominant neurotrophic factor in the brain, indispensable for neuronal growth, synaptic development, neuronal repair, and hippocampal neuroplasticity. Among its genetic variants, the BDNF Val66Met polymorphism is widespread in the population and has been associated with the onset and aggravation of diverse pathologies, including metabolic conditions like obesity and diabetes, cardiovascular ailments, cancer, and an array of psychiatric disorders. Psychiatric disorders constitute a broad category of mental health issues that influence mood, cognition, and behavior. Despite advances in research and treatment, challenges persist that hinder our understanding and effective intervention of these multifaceted conditions. Achieving and maintaining stable body weight is pivotal for overall health and well-being, and the relationship between psychiatric conditions and body weight is notably intricate and reciprocal. Both weight gain and loss have been linked to varying mental health challenges, making the disentanglement of this relationship critical for crafting holistic treatment strategies. The BDNF Val66Met polymorphism's connection to weight fluctuation in psychiatric patients has garnered attention. This review investigated the effects and underlying mechanisms by which the BDNF Val66Met polymorphism moderates body weight among individuals with psychiatric disorders. It posits the polymorphism as a potential biomarker, offering prospects for improved monitoring and therapeutic approaches for mental illnesses.

## 1. Introduction

Brain-derived neurotrophic factor (BDNF) is an essential protein within the central nervous system, fundamental to the processes of neuronal development, survival, and synaptic plasticity. Deviations in BDNF expression are linked to the pathogenesis and therapeutic responses of various mental, behavioral, and neurodevelopmental disorders, such as schizophrenia, eating disorders, depression, substance addiction, as well as other forms of mental and cognitive impairment [1]. Additionally, aberrant BDNF levels have been implicated in broader health issues, potentially including cancer, cardiovascular diseases, metabolic disorders, age-related diseases, immune system disorders, etc. [2]. BDNF exerts its effects primarily through the activation of the tropomyosin receptor kinase B (TrkB). Upon binding to TrkB, BDNF activates intracellular signaling cascades, such as phosphatidylinositol 3-kinase (PI3K)/Akt, mitogen-activated protein kinase (MAPK), and phospholipase C (PLC) pathways, which are involved in cell survival, cell differentiation, and synaptic plasticity [3].

The BDNF gene is located on the p13 band of chromosome 11, encompassing 11 exons and 9 functional promoters [4]. These promoters are responsible for initiating the transcription of various BDNF mRNA isoforms, all of which translate into an identical BDNF protein. Noteworthy mutations in this gene include rs6265, rs11030101, rs12291186, rs7934165, rs11030104, rs1519480, rs8192466, rs539177035, and rs551669106 [5]–[7]. The rs6265 single nucleotide polymorphism (SNP), commonly known as Val66Met, is a prevalent genetic variation within the human BDNF gene that interferes with the typical release of BDNF through the regulated secretory pathway. Consequently, this disruption in BDNF signaling, as influenced by rs6265, is implicated in contributing to a spectrum of diseases, encompassing obesity and metabolic syndrome, cardiovascular health, cancer, as well as neurological and psychiatric disorders, all of which share similarities with the pathology observed in cases of aberrant BDNF expression. Interestingly, multiple studies indicated that the Val66Met polymorphism was significantly involved in body weight changes in patients with psychiatric and behavioral disorders [8]–[10]. Maintaining a stable body weight is widely recognized as a critical component of overall health and well-being. Therefore, a deeper understanding and effective management of weight variations in patients with mental health disorders are paramount for delivering holistic care and improving health outcomes across both physical and mental dimensions. Some research posits that weight changes observed in psychiatric patients may be tied to the individual's self-perception of their mental illness, emotional fluctuations [11], hormonal imbalances [12],[13], and the side effects of psychiatric medications [14], all of which can potentially alter eating behaviors and metabolic state. To date, the precise molecular mechanisms driving weight gain or loss among psychiatric patients remain elusive. Building on this comprehensive overview, the objective of this article is to enhance our understanding of how the BDNF Val66Met polymorphism relates to weight variations in psychiatric patients, with the goal of informing the development of tailored treatment strategies and improving patient outcomes.

## 2. The BDNF Val66Met polymorphism is a genetic variation related to various health conditions linked with the wide-ranging biological roles of BDNF

The BDNF Val66Met polymorphism, designated as rs6265, represents a frequently occurring genetic variation within the BDNF gene. The specific SNP is located in the pro-domain of the BDNF protein at codon 66, which is part of the 5′ pro-region encoded by exon V. The polymorphism arises from a single nucleotide substitution where guanine (G) is replaced by adenine (A). Consequently, this nucleotide exchange triggers an amino acid alteration from valine (Val) to methionine (Met) at the 66th position of the BDNF precursor protein, leading to its designation as “Val66Met” [15],[16]. Structurally, the Val66Met polymorphism does not affect the mature BDNF protein directly but rather influences the intracellular trafficking and processing of proBDNF to mature BDNF [17]. Given BDNF's pivotal role in a multitude of physiological processes, this genetic variation has the potential to influence normal physiological functions considerably.

### 2.1. Neurodegenerative diseases and psychiatric conditions

Undoubtedly, the BDNF Val66Met polymorphism, also playing a crucial role in neuronal development, differentiation, and plasticity, is significantly associated with abnormal neurological function, which is notably linked to an increased susceptibility to neurodegenerative diseases and psychiatric disorders [18]. For instance, this polymorphism has been observed to modify hippocampal volume and amygdala-cortical connectivity, both of which are pivotal for memory and learning, as well as mood regulation [19].

A multitude of studies have been conducted to assess the impact of this genetic variation on neurodegenerative conditions such as multiple sclerosis (MS), Alzheimer's disease (AD), and Parkinson's disease (PD). Although the effects of the BDNF Val66Met polymorphism in MS, AD, and PD patients have yielded mixed outcomes, emerging evidence suggests that this genetic variant may have significant correlations with the disease's progression, cognition ability, learning, and memory function [20]. For example, this genetic variant showed a protective effect against hippocampal atrophy and cognitive decline in MS patients [21]. In the case of AD, the Met66 allele may contribute to the AD progression, potentially moderating memory impairment and hippocampal atrophy in early-stage AD patients who exhibit high levels of amyloid-beta (Aβ) and phosphorylation of tau181 [20],[22],[23]. Meanwhile, among PD patients, Met allele carriers are more prevalent than in the healthy population and exhibit significantly more pronounced deficits in mood/anxiety and psychotic symptoms compared to val allele carriers [20],[24]. Concurrently, a broad spectrum of research demonstrates that the BDNF Val66Met polymorphism is implicated in heightened vulnerability to psychiatric disorders, which span a diverse array of mental health challenges impacting mood, cognition, and behavior. The range of conditions associated with this genetic variation includes anxiety disorders, obsessive-compulsive disorder (OCD), depression, bipolar disorder, schizophrenia, eating disorders, as well as alcohol and substance-use disorders. Empirical evidence largely indicates that individuals with the Met/Met genotype tend to exhibit higher PANSS (Positive and Negative Syndrome Scale) positive scores in schizophrenia, suggesting more severe symptomatic manifestations. Moreover, Met/Met homozygosity has been correlated with diminished cognitive performance and reduced serum BDNF levels [25]. With respect to major depressive disorder (MDD), the Val66Met polymorphism has been associated with the illnesses, with the presence of the Met allele in patients correlating with a more favorable response to antidepressants [26]. Current studies also reveal that possession of the Met allele may elevate the risk for generalized anxiety disorder compared to control groups [27]. In the context of OCD, carriers of the “A” allele (corresponding to the Met variant) have been linked to lower symptom dimension scores, hinting at a potential protective role of the Met allele in the disorder [28]. These findings collectively underscore the significant influence of the BDNF Val66Met polymorphism on the etiology and therapeutic outcomes of various neurodegenerative diseases and psychiatric conditions.

### 2.2. Cancers

Numerous studies have established an association between the BDNF Val66Met polymorphism and the incidence of cancer. For instance, research by Li et al. highlighted an elevated risk of bladder cancer linked to the Met66 variant. This increase in risk is thought to be modulated via the miR-146b/CRK/Akt signaling pathway, which significantly influences cancer cell growth and programmed cell death [29]. Additionally, the Val66Met polymorphism has been connected to psychological alterations in individuals diagnosed with cancer. Lan et al. found a notable link between the T/C and T/T genotypes at rs6265 and the manifestation of depressive symptoms in Chinese patients with early-stage breast cancer [30]. In a similar vein, research by Yap et al. indicated that Met allele carriers were somewhat shielded from subjective cancer-related cognitive impairment (CRCI) post-chemotherapy, with improvements observed especially in multitasking and memory functions. This ameliorative effect was also apparent in persistent CRCI, where it alleviated challenges related to mental sharpness and the ability to juggle multiple tasks [31]. These insights point to the potential role of the BDNF Val66Met polymorphism as a dual-purpose biomarker, facilitating not only the detection of cancer but also the identification of patients at a heightened risk for ensuing psychological difficulties.

### 2.3. Cardiovascular diseases and cardiometabolic disorders

Beyond its well-documented roles, the BDNF Val66Met polymorphism has also been implicated in a range of cardiovascular diseases, inclusive of cardiometabolic disorders, coronary artery disease (CAD), and cardiomyopathy associated with Duchenne muscular dystrophy (DMD). Clinical investigations have identified a significant relationship between the frequency of the BDNF Val66Met genotype and the occurrence of unstable angina pectoris (UAP). Notably, the Met/Met genotype appears to confer a protective effect against the development of UAP, and this remains the case even when traditional CAD risk factors are taken into account [32]. Moreover, the Val66Met polymorphism is known to influence myocyte contractility. While this genetic variation is associated with compromised skeletal muscle functions, it paradoxically relates to enhanced cardiac function in DMD patients. This dualistic influence underscores the intricate role that the Val66Met polymorphism plays in the dynamics of muscle function [33]. Interactions between genes and diet are increasingly recognized as significant in shaping the risk profile for cardiometabolic diseases. Research indicates that the BDNF Val66Met polymorphism may modulate cardiometabolic markers, such as the dietary insulin index and dietary insulin load, particularly in individuals with diabetes. The Met allele, in this context, is posited to have a protective effect, potentially exerting a favorable influence on cardiometabolic parameters by mediating dietary influences [34],[35].

## 3. A notable correlation exists between BDNF Val66Met polymorphism and weight fluctuations in patients suffering from psychiatric disorders

Currently, most studies have suggested that BDNF may be a major factor in the development of psychiatric disorders [2]. Genetic mutations have altered the efficacy of BDNF signaling, the Val66Met polymorphism is also associated with impaired neurocognitive function in healthy adults and has been identified as a risk locus for a range of neuropsychiatric disorders. BDNF is involved in the control of glucose, lipid, and antioxidant metabolism and this is considered to be a signal of anaerobicity in food intake control centers [36],[37]. Thus, BDNF is considered not only as a neurotrophic factor but also as a metabolic factor. Significantly, fluctuations in body weight are a frequent occurrence among patients with disorders linked to the BDNF Val66Met polymorphism, particularly within the realm of psychiatric conditions. Studies indicate that the BDNF Val66Met polymorphism may influence both weight gain and weight loss, predominantly through its role in the regulation of appetite and energy balance within the brain [38]. Additionally, this genetic variation may alter how patients react to psychiatric medications, which frequently have significant weight gain as a side effect [39]. Moreover, the Val66Met variant is potentially linked to an increased risk of metabolic syndrome in individuals with psychiatric disorders—an ailment marked by central obesity and insulin resistance [40]. This review primarily explores the influence of the BDNF Val66Met polymorphism on body weight regulation among patients with a range of psychiatric disorders, including schizophrenia, anorexia nervosa, and bulimia nervosa (eating disorders), bipolar disorder, major depressive disorder, obsessive-compulsive disorder (OCD) and related conditions, post-traumatic stress disorder (PTSD), as well as substance-related and addictive disorders.

### 3.1. Schizophrenia

Schizophrenia is a multifaceted and chronic medical condition that impairs an individual's cognitive, emotional, and behavioral clarity. It presents with a diverse spectrum of psychological symptoms, positioning it as one of the most intricate challenges within the realm of mental health, affecting approximately 1% of people worldwide. The precise etiology of schizophrenia remains elusive; however, it is generally considered to arise from a complex interplay of genetic predispositions, neurobiological variations, and environmental influences [41]. Contemporary research has increasingly pointed toward BDNF Val66Met polymorphism as playing a critical role in weight changing during the pathogenesis of schizophrenia.

A robust link has been established between the BDNF Val66Met polymorphism and body mass index (BMI) among chronically ill and medicated male schizophrenia patients [42]. Those with the Met/Met genotype displayed a greater average BMI compared to their Val/Val genotype counterparts. These findings suggest that BDNF gene variations may predispose male individuals with schizophrenia, especially those undergoing long-term antipsychotic treatment, to increase weight gain. The study further revealed that all three BDNF Val/Met genotypes correlated positively with a rise in mean BMI, with the Met/Met genotype group experiencing a notably higher surge in BMI than the Val allele carriers. The influence of genotype on BMI was pronounced among male patients, but not the female cohort. Thus, BDNF gene variations could represent a risk factor for weight escalation in the male schizophrenia population [42]. Interestingly, while serum BDNF levels were significantly reduced in patients overall, the levels did not differ markedly between the allele groups. Men's serum BDNF levels appeared unaffected by genotype, whereas women with the Met/Met genotype exhibited significantly lower BDNF levels than those with the Val allele. A negative correlation between serum BDNF levels and BMI was identified, but this was exclusive to women [43].

The relationship between the BDNF-rs6265 genotype and weight variation in patients with schizophrenia, along with the underlying mechanisms, remains to be fully elucidated. However, the evidence of sex-specific effects of BDNF polymorphisms provides a promising direction for future research into the genetic underpinnings of weight fluctuations in this context.

### 3.2. Eating disorders including anorexia nervosa and bulimia nervosa

Anorexia nervosa (AN) is a debilitating eating disorder characterized by an alarmingly low body weight, an intense fear of weight gain, and a distorted body image. Those affected by anorexia are often preoccupied with controlling their weight and shape, going to extreme lengths that can markedly disrupt their lives. As a result, AN is noted for having the highest mortality rate among psychiatric conditions and is frequently accompanied by both acute psychiatric and medical comorbidities. Bulimia nervosa (BN), another grave eating disorder, involves recurrent episodes of binge eating followed by compensatory measures, often referred to as purging, in an attempt to counteract the caloric intake from binges. Compensatory measures of BN include strict dieting and extreme exercise. AN and BN predominantly impact women and present as complex psychiatric illnesses influenced by a myriad of genetic and environmental factors [44]. Despite their prevalence and severity, our understanding of the intricacies of AN and BN remains considerably limited.

Emerging studies in rodent models have revealed that modifications in BDNF/TrkB signaling can lead to the development of bulimic and obese phenotypes, while pharmacologically induced elevation of BDNF levels results in decreased food consumption [45]. These observations suggest that variations in BDNF may contribute to a vulnerability to eating disorders in humans. Notably, the Val66Met (G196A) polymorphism in the BDNF gene has been implicated in the genetic underpinnings of such disorders. For instance, the presence of the Met allele in the BDNF gene has been correlated with amplified hunger-driven reward responses observed in patients with AN [8]. Current research has indicated that the Val66Met may play a role in promoting weight gain in women diagnosed with BN, potentially through gene-gene interactions. A study by Kaplan et al. highlights this interaction by demonstrating that individuals with BN who possess both the hypofunctional 7R allele of the dopamine receptor D4 (DRD4) and the Met66 allele of BDNF exhibited a significantly higher maximal BMI compared to those in other gene-gene interaction cohorts, such as carriers of the 7R allele of DRD4 paired with the 270C/T polymorphism of BDNF [46].

### 3.3. Bipolar disorder and major depressive disorder

Bipolar disorder (BD), commonly referred to as manic-depressive illness, is a complex psychiatric condition marked by severe fluctuations in mood, ranging from episodes of elevated moods, known as mania or hypomania, to periods of intense depression. The origins of BD are not entirely understood, but it is believed that a combination of genetics, environment-altered brain structure, and chemistry may play a role. A notable impact of BD is on lifestyle and physiological functions—it can disrupt sleep patterns, impair judgment, alter behavior, cloud thinking, and influence energy levels, often leading to variations in body weight [47]. The correlation between specific BMI phenotypes and the presence of the BDNF Val66Met polymorphism in patients with BD was also studied. Research indicates that carriers of the Val allele could be at an increased risk of being overweight and obese, while the Met allele might be associated with a lower risk of these conditions in the BD population [40],[48]. Furthermore, the BDNF Val66Met polymorphism has been linked to antipsychotic-induced weight gain among BD patients. Specifically, Met66 allele carriers demonstrate notable elevations in the triglycerides-to-high-density lipoprotein cholesterol (HDL) ratio and in log-triglycerides after three or six months of treatment. This ratio is a critical indicator of metabolic syndrome, reflecting insulin resistance [42]. The association of the BDNF Val66Met polymorphism with increased BMI is consistent with earlier findings in schizophrenia patients and suggests that the BDNF Val66Met genotype may be a significant risk factor for obesity and metabolic disturbances in patients with BD, particularly those undergoing antipsychotic treatment.

Major depressive disorder (MDD), also known simply as depression, is characterized by a depressed mood, a loss of interest or pleasure in activities, changes in appetite, trouble sleeping or oversleeping, energy loss, feelings of worthlessness or excessive guilt, difficulty thinking, concentrating, or making decisions, and thoughts of death or suicide. About 4.4% of the world's population is affected by depression, and about 1 million people die by suicide each year. The exact cause of MDD is unknown, but genetics seems to be involved [49]. Although a lot of evidence indicates that BDNF plays an important role in MDD, new research revealed that although a tendency toward an interaction was found in the Radiant sample, no difference was found in BMI depending on the Val66Met genotype and no interaction between this polymorphism and MDD in relation to BMI was found with meta-analysis [50].

However, research has demonstrated that there is a notably high occurrence of major depression among individuals with type 2 diabetes (T2DM), coinciding with diminished BDNF levels—circumstances that could be linked to the BDNF Val66Met polymorphism [51]. Furthermore, research suggests that the BDNF Val66Met polymorphism may influence BMI in diabetic individuals by affecting dietary habits and safeguarding against adverse metabolic indicators. Regrettably, current research does not conclusively establish a relationship between the BDNF Val66Met polymorphism and body weight in patients with coexisting diabetes mellitus and depression.

### 3.4. Obsessive-compulsive disorder (OCD) and related disorders

Obsessive-compulsive disorder (OCD) is a mental health condition marked by ongoing, intrusive thoughts (obsessions) and ritualistic behaviors or mental routines (compulsions) that an individual feels compelled to perform. Conditions related to OCD encompass a spectrum of disorders sharing core features with OCD, such as body dysmorphic disorder, hoarding disorder, hair-pulling disorder, and excoriation disorder, among others. Both OCD and its related disorders can profoundly disrupt an individual's daily life, affecting their professional performance, social engagements, and overall well-being [52]. Hoarding, often considered a distinct entity within the spectrum of OCD-related conditions, is a persistent and debilitating disorder that constitutes a considerable public health concern. Evidence suggests that the Val/Val genotype is associated with hoarding classifications, heightened severity of hoarding behaviors, and increased BMI levels. These findings underscore that individuals with the Val/Val genotype have a considerably higher risk of developing a hoarding disorder compared to Met allele carriers. They also suggest a more robust correlation between the Val allele and obesity, with individuals carrying the Val/Val genotype having a higher propensity for obesity [53]. This research may unveil a novel pathway linking hoarding behaviors and BMI through the BDNF Val66Met polymorphism.

### 3.5. Substance-related and addictive disorders

Substance-related and addictive disorders constitute a spectrum of mental health conditions characterized by the excessive consumption of substances such as alcohol, drugs, and tobacco, which lead to considerable impairment or distress. These disorders are characterized by a range of harmful behaviors and symptoms, including substance dependence and abuse, and substance and behavioral addiction [54].

Research has revealed a notable link between addictive disorders and fluctuations in body weight. Individuals grappling with addictions, ranging from alcoholism, smoking, drug use, digital game addiction (DGA), smartphone dependency, and beyond, often engage in other unhealthy lifestyle practices, which invariably contribute to changes in their body mass index (BMI) [55]–[58]. BMI can predict internet addiction, Facebook addiction, and smoking in certain eating disorder groups [59]. Moreover, it has been established that the Val66Met variation exerts an influence on the predisposition to substance-related behaviors, such as smoking and drug use. For instance, studies have shown that the presence of the Met/Met genotype and the Met allele occurs with greater frequency in individuals who are current or former smokers, in contrast to those who have never smoked [60]. Similarly, a higher occurrence of the BDNF 66Val homozygote has been observed in individuals with drug addictions as opposed to the general population [61]. Despite these findings, the potential correlation between BDNF polymorphisms and body weight changes in those suffering from addictive disorders has yet to be investigated.

### 3.6. Post-traumatic stress disorder

Post-traumatic stress disorder (PTSD) is a mental health disorder that is triggered by experiencing or witnessing a terrifying event. Symptoms may include flashbacks, nightmares, severe anxiety, and uncontrollable thoughts about the event. Individuals with PTSD may also experience emotional numbness and avoidance of places, people, and activities that are reminders of the trauma [62].

Extensive research suggests that PTSD, frequently observed in veterans and individuals who have endured catastrophic events such as the World Trade Center attack and the Fukushima nuclear disaster, is notably linked to higher instances of obesity and expedited weight gain [63]–[65]. Within populations affected by PTSD, particularly veterans, there is a notable connection between the BDNF Val66Met polymorphism and cognitive performance. Research by Havelka and others suggests that the presence of the BDNF Val66Met A allele, in contrast to the GG genotype, is associated with reduced short-term visual memory and attention, essential components of executive function, in veterans grappling with PTSD. In addition, further investigation has revealed significant interplay between carriers of the Met allele and the likelihood of PTSD, with implications for both objective and subjective cognitive abilities in U.S. military veterans [66]. Moreover, studies have uncovered a substantial genetic predisposition toward suicidal tendencies in PTSD sufferers with a background of childhood physical abuse, specifically linked to this BDNF gene variation. However, following the 27-F earthquake in Chile in 2010, researchers observed no significant differences in the Val66Met polymorphism among individuals who developed post-earthquake PTSD [67].

Additionally, the body of research examining the potential connection between weight fluctuations in PTSD patients and the BDNF gene polymorphism remains scant. However, one study has demonstrated that specific BDNF variants, namely Val66Met and C270T, do not show a significant association with metabolic indices in veterans with PTSD. This indicates that these particular genetic variations might not influence the susceptibility to metabolic disorders in individuals with PTSD, thereby suggesting that significant shifts in body mass index are unlikely to be attributed to these polymorphisms [68].

## 4. Conclusions

Psychiatric disorders represent a vast range of mental health issues that affect a person's cognition, emotions, mood, and behavior. Although significant strides have been made in their diagnosis and treatment, persistent challenges underscore the necessity for ongoing research and innovation in the field. These disorders span from prevalent conditions such as anxiety and depression to more intricate pathologies like schizophrenia and bipolar disorder. The origins of these disorders are heterogeneous and multifaceted, encompassing a dynamic interplay of environmental influences, psychological stress, biochemical imbalances, and genetic susceptibilities.

Brain-derived neurotrophic factor (BDNF) serves as a pivotal molecule within the central nervous system, fostering neuron survival, growth, and differentiation. It is also instrumental in synaptic plasticity, a key process for learning and memory functions. Variations in BDNF levels have been observed across a range of psychiatric disorders, with its regulatory role intimately connected to both the underlying pathophysiology and therapeutic interventions of these ailments. Genetic variations have notably altered BDNF signaling efficiency, such as the BDNF Val66Met polymorphism, a single nucleotide polymorphism in the BDNF gene, which results in the substitution of methionine (Met) for valine (Val) at the 66th codon. This genetic alteration has critical implications. Body weight is a fundamental marker of overall health and maintaining a balanced body weight is essential for averting various health complications, including those affecting mental health. The relationship between body weight and psychiatric disorders is intricate, with current research efforts aimed at deciphering these associations to enhance treatment and preventative measures for those impacted by psychiatric disorders, modulating BDNF's influence on neuroplasticity and cognition.

This review has revealed a strong association between the BDNF Val66Met polymorphism and weight fluctuations in patients with psychiatric disorders, specifically including schizophrenia, eating disorders (such as anorexia nervosa and bulimia nervosa), bipolar disorder, major depressive disorder, obsessive-compulsive disorder (OCD), and related conditions. But the direct link between BDNF polymorphism and body weight alterations in individuals with addictive disorders and PTSD is yet to be explored. In an effort to substantiate the link between BDNF polymorphism and body weight in patients with psychiatric disorders, we utilized cross-tabulations and conducted Pearson's chi-squared tests on data extracted from seven studies encompassed in five scholarly publications, including references [50],[69]–[72]. These analyses confirmed a significant association (*P* < 0.05) between the BDNF Val66Met polymorphism and weight shifts in individuals with psychiatric disorders who share the polymorphism, as evidenced by their similar average BMIs. Additionally, the BDNF Val66Met polymorphism also influences the trajectory of psychiatric disorders by affecting the development of particular brain regions, serum BDNF levels, interactions with other genes, and dietary habits. Thus, this review is instrumental in advancing our comprehension of the mechanisms that connect body weight and mental health, proposing the BDNF Val66Met polymorphism as a prospective biomarker for monitoring body weight in psychiatric conditions, and in informing risk assessments and therapeutic strategies in psychiatry. Nevertheless, the precise mechanisms through which Val66Met impacts weight changes in individuals with psychiatric disorders remain elusive. This knowledge gap provides pivotal guidance for future research and charts a novel course for subsequent inquiries.

## Use of AI tools declaration

The authors declare they have not used Artificial Intelligence (AI) tools in the creation of this article.
